# National Trends in Telehealth Utilization, 2020–2023: Post-Pandemic Trends from the Medical Expenditure Panel Survey

**DOI:** 10.3390/healthcare14030331

**Published:** 2026-01-28

**Authors:** Jaewhan Kim, Elise V. Bailey, Steven Hayworth, Chathuri Illapperuma-Wood, Rachel Weir, Aaron Fischer, Peter Weir

**Affiliations:** 1 Department of Physical Therapy, University of Utah, 520 Wakara Way, Salt Lake City, UT 84108, USA; 2 Department of Population Health Sciences, University of Utah, 295 Chipeta Way, Williams Building, Salt Lake City, UT 84108, USA; elise.bailey@utah.edu; 3 Medical Group Population Health, University of Utah, 50 North Medical Drive, Salt Lake City, UT 84132, USA; steven.hayworth@hsc.utah.edu (S.H.); peter.weir@utah.edu (P.W.); 4 Department of Educational Psychology, University of Utah, 1721 Campus Center Drive SAEC 3220, Salt Lake City, UT 84112, USA; chathuri.illapperuma@utah.edu (C.I.-W.); aaron.fischer@utah.edu (A.F.); 5 Department of Psychiatry, University of Utah, 501 Chipeta Way, Salt Lake City, UT 84108, USA; rachel.weir@hsc.utah.edu

**Keywords:** telehealth, health care delivery, post-pandemic period, medical expenditure panel survey

## Abstract

**Background/Objectives:** U.S. telehealth utilization increased substantially during the COVID-19 pandemic; however, post-pandemic utilization patterns and associated characteristics remain unclear. This study examined national trends in telehealth use and identified factors associated with its utilization from 2020 to 2023. **Methods:** Data from the Medical Expenditure Panel Survey (2020–2023) Office-Based Medical Provider Visits and Outpatient Visits files were used to identify national telehealth use. Descriptive statistics and generalized linear regression were used to examine telehealth utilization, visit type, service type, and potential predictors of utilization. **Results:** The proportion of U.S. health care visits made via telehealth increased sharply from 2020 (1.84%; 95% CI, 1.67–2.01) to 2021 (4.53%; 95% CI, 4.11–4.94) and then stabilized through 2023. The proportion of the U.S. population with at least one telehealth visit followed a similar trend (7.15% in 2020; 12.09% in 2021; 12.05% in 2022; 12.12% in 2023). Telehealth visits were primarily for outpatient care and were most commonly used for mental health services (4.20% in 2021; 4.13% in 2022; and 4.18% in 2023). Sex, health insurance status, age, and family income were significant predictors of telehealth utilization. **Conclusions:** Pandemic-related increases in telehealth use have persisted beyond the COVID-19 period. Continued support from health care systems and policymakers is necessary to sustain and expand access to telehealth services.

## 1. Introduction

Telehealth has become a vital component of health care delivery, particularly since the COVID-19 pandemic. Although telehealth has existed in the U.S. for decades, utilization was rare before 2020. One study found that 0.020 in 1000 privately insured patients used telehealth in 2005, and 6.57 in 1000 patients did so in 2017 [1]. In January 2020, telehealth utilization represented less than 1% of claims to private insurers, and there were major restrictions on the types of telehealth that would be reimbursed [2]. The pandemic resulted in an unprecedented increase in telehealth utilization. Previous restrictions were lifted, allowing patients to be seen in their homes, and the Drug Enforcement Administration (DEA) temporarily allowed for prescription of a controlled substance via telehealth. Estimates of peak utilization range between 15% and 50% of visits, depending on the population and time period examined (there was significant intra-year variation during 2020) [3,4,5,6]. In 2022, as many as 39.3% of U.S. adults had past-year telehealth use [7]. The primary reasons patients cite for using telehealth post-pandemic are minor or acute illness, chronic disease management, mental health or substance use, and annual exams [8,9]. During the pandemic, the greatest telehealth uptake was among the mental health, gastroenterology, and endocrinology specialties [10].

Telehealth utilization is related to sex, race/ethnicity, education, health insurance type, and marital status: women, non-Hispanic white individuals, college graduates, the insured, married individuals, and people with chronic health conditions are more likely to use it than their peers [3,8,11,12,13,14,15]. There is disagreement in the literature about whether age, socioeconomic status, or rural residence are related to telehealth utilization post-pandemic [7,8,9,11,14,16,17]. Racial minorities and older adults may be more likely to use audio-only telehealth than video-based modalities [12,18].

Despite the growing body of research on telehealth use, national-level studies examining trends in telehealth utilization remain limited. Many studies examining telehealth utilization have restricted samples as a result of using claims or electronic medical record data in their analyses [19,20,21,22]. Relatively few studies examine telehealth utilization using nationally representatively survey data [5,6,7,20]. To our knowledge, none examine trends across years. These data choices may contribute to variability in utilization estimates and inconsistent findings regarding predictors of telehealth use. To address these gaps, this study used nationally representative data from the Medical Expenditure Panel Survey (MEPS) from 2020 to 2023 to examine national trends in telehealth use. This study assessed differences by visit type (office-based vs. outpatient) and service type (e.g., mental health, chronic disease management), and identified factors associated with telehealth utilization.

## 2. Materials and Methods

**Data.** This study used 2020–2023 MEPS data. MEPS is a nationally representative survey of the civilian, noninstitutionalized U.S. population [23,24]. Since telehealth use has expanded during the pandemic, MEPS has collected data on telehealth visits over time. Telehealth visits were captured in both office-based and outpatient settings using the Office-Based Medical Provider Visits Files and Outpatient Visits Files. MEPS defines office-based visits as visits to a doctor’s or group practice office, medical clinic, managed care plan or HMO center, neighborhood/family/community health center, surgical center, rural health clinic, company clinic, school clinic, walk-in urgent centers, VA facility, or laboratory/X-ray facilities [25]. Outpatient visits are defined as visits to a hospital outpatient department, including any unit or facility that provides care which does not require overnight hospitalization [26]. These visit files were linked to the Full Year Consolidated Data Files, which contain information on demographics, health conditions, access to care, quality of care, and employment status [24]. This study is not considered human subjects research and therefore does not require IRB review.

**Subjects.** This study included all individuals (≥0 years old) surveyed in MEPS from 2020 through 2023, with no exclusions.

**Outcomes.** The primary outcome was telehealth use rates over time (2020–2023), calculated as:

Telehealth rate (%) = (telehealth visits/total visits) × 100

In addition to visit-level telehealth rates, person-level telehealth utilization was examined. Two subject-level outcomes were analyzed: any telehealth use (yes/no) and the number of telehealth visits among users. These outcomes were used to examine trends and identify factors associated with telehealth adoption and intensity of use.

The definition of ‘telehealth’ has varied across studies [27,28,29,30,31]. In this study, MEPS defines telehealth as a remote visit (via phone, video, or other modalities) between a patient and a health care provider for the purpose of diagnosis or treatment.

**Covariates.** Telehealth use rates were analyzed by visit type (office-based vs. outpatient), service type (check-up, diagnosis, emergency, psychotherapy, follow-up, and other services), age group (0–17, 18–64, and ≥65 years), sex (male vs. female), race/ethnicity (non-Hispanic White, Hispanic, non-Hispanic Black, non-Hispanic Asian, and non-Hispanic Others), family income (poor, near poor, low, middle, and high income) and type of health insurance (private, public, or uninsured).

**Statistical Approach.** Because MEPS is a nationally representative survey, all analyses were weighted to reflect the U.S. population [32,33]. Summary statistics, including means, standard deviations (SDs), and percentages, were used to describe population characteristics. Chi-square tests were applied for categorical variables and linear regression for continuous variables to assess differences across years. Given that more than 80% of individuals had no telehealth visits and to examine the intensity of telehealth use among those who did use telehealth, a survey-weighted two-part modeling approach was employed to identify factors associated with both telehealth use and the number of telehealth visits. In the first part, logistic regression was used to model the probability of any telehealth use (telehealth use vs. no telehealth use). In the second part, negative binomial regression was used to model the number of telehealth visits among individuals with at least one telehealth encounter, accounting for the count nature and overdispersion of the outcome variable. Results from the first part are presented as odds ratios (ORs), and results from the second part are presented as incidence rate ratios (IRRs). As robustness checks, weighted zero-inflated negative binomial (ZINB) regression was estimated, and additional sensitivity analyses excluding the top 1% and top 5% of visit utilizers were conducted to assess whether results were driven by individuals with high levels of health care use. To obtain valid standard errors and *p*-values that account for the complex sampling design of MEPS, all analyses incorporated survey weights and design variables, and standard errors were estimated using the Taylor-series linearization method, as recommended by MEPS [34].

*p*-Values less than 0.05 were considered statistically significant. This study follows STROBE guidelines [35].

## 3. Results

Over 300 million people were represented in the analysis. The average age was about 40 (SD = 23) years old, and 51% were female. Approximately 59% were Non-Hispanic White, and 19% were Hispanic (Appendix A, Table A1). The average number of office and outpatient visits was around 7 visits (SD = 13 to 14 visits) and the average number of telehealth visits for office and outpatient visits was around 0.7 (SD = 0.2 to 0.7 visits) across the years (Table 1).

Table 2 shows telehealth use by year. Between 2020 and 2023, telehealth utilization increased from 2020 to 2021 and then stabilized in subsequent years. The telehealth rate rose from 1.8% (95% CI, 1.7–2.0) in 2020 to 4.5% (95% CI, 4.1–4.9) in 2021, remained similar in 2022 (4.5%), and slightly increased in 2023 (4.8%). The proportion of the U.S. population with at least one telehealth visit followed a similar pattern, increasing from 7.2% in 2020 to 12.1% in 2021, 12.1% in 2022, and 12.1% in 2023.

Table 3 shows telehealth utilization by visit type. Telehealth use increased from 2020 to 2021 and remained relatively stable through 2023 for both office and outpatient visits. The telehealth rate for office visits rose from 1.9% in 2020 to about 4.5–4.8% during 2021–2023, while the proportion of the population with a telehealth office visit nearly doubled from 4.9% to around 8–9% over the same period. Telehealth use for outpatient visits was consistently higher than for office visits, increasing from 8.6% in 2020 to 15.2% in 2021–2022, followed by a modest decline to 12.7% in 2023. The proportion of the population with an outpatient telehealth visit followed a similar pattern, peaking in 2021 (5.2%) and slightly decreasing thereafter.

Patterns varied by service type (see Table 4). Psychotherapy consistently showed the highest telehealth utilization, increasing from 1.2% in 2020 to 4.2% in 2021, 4.1% in 2022, and 4.2% in 2023. Conversely, telehealth use for check-up and diagnostic visits rose sharply between 2020 and 2021, then slightly declined or leveled off in 2022–2023. Telehealth use for emergency visits remained very low (<0.1%) across all years.

Visit-level telehealth rates by demographic characteristics were examined across years and did not change substantially between 2021 and 2023 (Appendix B, Table A2).

The regression analysis identified factors significantly associated with telehealth use, including time (see Table 5). Compared with 2020, the odds of any telehealth use were 102% higher in 2021 (OR = 2.02, *p* < 0.01), 86% higher in 2022 (OR = 1.86, *p* < 0.01), and 81% higher in 2023 (OR = 1.81, *p* < 0.01). Among individuals who used telehealth, the number of telehealth visits was 39% higher in 2021 (IRR = 1.39, *p* < 0.01), 36% higher in 2022 (IRR = 1.36, *p* < 0.01), and 50% higher in 2023 (IRR = 1.50, *p* < 0.01) compared with 2020. Compared with children aged 0–17 years, adults aged 18–64 years had 133% higher odds of using telehealth (OR = 2.33, *p* < 0.01), and adults aged ≥65 years had 81% higher odds of telehealth use (OR = 1.81, *p* < 0.01). However, among telehealth users, adults aged ≥65 years had 46% fewer telehealth visits than children (IRR = 0.54, *p* < 0.01), while no significant difference in visit counts was observed for adults aged 18–64 years (IRR = 0.97, *p* = 0.50). Females had 37% higher odds of any telehealth use compared with males (OR = 1.37, *p* < 0.01). Among telehealth users, the number of telehealth visits did not differ significantly by sex (IRR = 0.97, *p* = 0.31). Compared with non-Hispanic White individuals, Hispanic individuals had 17% lower odds of telehealth use (OR = 0.83, *p* < 0.01), non-Hispanic Black individuals had 23% lower odds (OR = 0.77, *p* < 0.01), and non-Hispanic Asian individuals had 19% lower odds (OR = 0.81, *p* = 0.01). No difference in telehealth adoption was observed for non-Hispanic individuals of other races (OR = 1.02, *p* = 0.88). Among telehealth users, non-Hispanic Black individuals had 10% more telehealth visits than non-Hispanic White individuals (IRR = 1.10, *p* = 0.03), whereas visit counts did not differ significantly for Hispanic (IRR = 1.04, *p* = 0.27), non-Hispanic Asian (IRR = 0.89, *p* = 0.11), or non-Hispanic other individuals (IRR = 1.00, *p* = 0.96). Results from the zero-inflated negative binomial model were similar to those from the negative binomial model, indicating that the findings were robust to alternative specifications accounting for excess zeros. Sensitivity analyses excluding the top 1% of individuals with high utilization (≥67 visits) and the top 5% of individuals with high utilization (≥32 visits) yielded results that were unchanged in direction, magnitude, and statistical significance.

## 4. Discussion

Telemedicine has great potential to increase health care access, especially for rural dwellers and patients who need specialty or culturally appropriate care [16,36,37,38,39,40,41,42,43,44,45,46]. However, issues related to reimbursement, adoption costs, digital literacy, and licensure requirements prevented its wide use until the pandemic [36,47,48,49,50,51], when emergency or temporary policy changes addressed many of those problems [49,52]. Previous research found that telehealth utilization increased dramatically during the pandemic and remained higher than pre-pandemic levels after the pandemic ended, but exact utilization rates and predictive factors have been unclear. By using nationally representative data from the MEPS (2020–2023), this study adds clarity.

This study found that the proportion of U.S. health care visits made via telehealth using telephone, video, or other modalities increased from 1.84% in 2020 to about 4.53% in 2021 and 2022, then slightly increased to 4.81% in 2023. These estimates—which include all payers—are roughly consistent with proportions derived from private claims in December of 2021, 2022, and 2023 (4.9%, 5.5%, and 4.9%, respectively) [53,54,55]. It is important, however, to note that the populations differ between our study and private claims data, and such comparisons are therefore of limited utility. In adjusted analyses, this study found that utilization was significantly higher in 2021–2023 than in 2020. However, adjusted utilization decreased somewhat between 2021 and 2022. Because our results are in terms of annual utilization and utilization rates changed rapidly during the early pandemic, they obscure April 2020’s peak telehealth utilization, which may have been as high as 50% that month [3]. Even given this caveat, our results show that at least some of the pandemic-related gains in telehealth utilization endured beyond that period and have remained relatively stable through the end of 2023.

The proportion of the U.S. population with at least one telehealth visit—as defined by MEPS—increased from 7.15% in 2020 to about 12.1% in subsequent years. This is far lower than estimates from the small number of previous nationally representative studies (between 39.3% and 50% of U.S. adults) [7,8,56]. The reasons for the large differences from prior studies are unclear. All these studies define telehealth use similarly, but other methodological differences between surveys may provide explanations. Our study includes children, and prior studies did not. Prior studies also relied on data from the annual Health Information National Trends Survey (HINTS) or from one-time surveys administered by study authors [7,8], whereas MEPS collects information from respondents five times over a two-year period [34]. HINTS respondents may have worse recall due to memory decay [57,58,59]. In addition, MEPS surveys ask respondents to provide specific information about each individual health care visit a respondent had, whereas the other surveys asked whether any telehealth visit was made in the past year [8,34,56,60]. The additional information required by MEPS, as well as its shorter recall periods, may help prevent forward telescoping, which is a major reason for overreporting [61,62]. These methodological differences may make MEPS estimates of telehealth use more reliable than those derived from other surveys. However, it is also possible that MEPS estimates underreport telehealth utilization; past validation studies have shown that MEPS respondents underestimate some kinds of health care utilization [63].

There were more telehealth visits, as defined by MEPS, over this period (2020–2023) for hospital outpatient visits than for office-based visits, in relative terms. It is not clear from our data or the literature why this may be. One potential hypothesis could be that this result is reflective of telehealth adoption patterns. Perhaps hospitals, given their extensive resources, are more likely than offices and clinics to adopt telehealth. At least one study has found that health system-owned clinics are more likely to adopt telehealth than those that are independently owned [64], which provides some limited evidence for this theory. Additionally, hospital outpatient visits may have higher patient acuity than office-based visits, and policy differences, including reimbursement rules, may also explain the differences we observed. It may also be the case that, because there are fewer hospitals than offices in the U.S., the travel distance to in-person care is greater and therefore more inconvenient for outpatient visits than office-based visits. Our findings around service type—that telehealth via telephone, video, or other modalities is most utilized for mental health care—are consistent with the literature [8,9,65].

Our findings that sex, race/ethnicity, and health insurance status predict telehealth use are also consistent with the literature [3,8,11,12,14]. Previous studies have presented conflicting results as to whether age and socioeconomic status are related to telehealth utilization post-pandemic [7,8,9,11,14,16,17]. This study found that age and family income were related to telehealth use, even when adjusting for other factors; middle-aged people (18–64) were more likely to use telehealth than other age groups, and people in high-income families were more likely to use it than those in poor families. While telehealth has the potential to entirely eliminate distance- and travel-related barriers to care and may reduce some disparities as a result, it is also possible that barriers to telehealth itself may exacerbate other health care access disparities or even create new ones. For example, not all people have access to sufficiently high-quality internet or have the digital literacy necessary for telehealth use. These barriers to telehealth are common among older adults, those with low socioeconomic status, racial/ethnic minorities, and rural residents [47,51,66,67,68]. This study’s findings that older adults, people in low-income families, and the uninsured were less likely than their peers to utilize telehealth only reinforce such concerns.

Our findings have implications for health care systems and policymakers. Telehealth is now an important and potentially permanent part of the health care landscape. Policymakers must find ways to support telehealth use—including audio-only telehealth—through reimbursement policy and licensure portability. Reimbursement parity is critical in the case of tele-mental health care, given the common use of telehealth for those services. The literature also suggests it is vital that systems continue to invest in patient and provider education to ensure high-quality delivery of services via telehealth modalities.

Despite the use of nationally representative survey data, this study has several limitations. First, data on outpatient and office visits are self-reported and may be subject to recall bias. Such bias could potentially affect estimates of telehealth use and trends over time. This limitation should be considered when interpreting the results and addressed in future studies using data sources with more detailed interview timing information. This limitation may be less profound in MEPS than in other surveys, as previously discussed. Future studies should validate MEPS estimates of telehealth utilization via linkages to claims data. Second, there are other important factors that may predict telehealth use but were not included in our analyses. This includes rurality, internet access, and digital literacy, which were not available in MEPS. Such variables may confound or mediate the relationships between sociodemographic factors and telehealth utilization. As a result, these estimates should be interpreted with caution. Future studies should endeavor to collect this information. The National Health Interview Survey includes measures of rurality and internet access that can be linked to MEPS data, although access to these linked data is restricted. To our knowledge, no nationally representative survey currently collects data on both telehealth and digital literacy. Government agencies should consider the addition of such questions to their surveys.

In addition, because the omission of important access-related variables (such as rurality and internet access) that constrain telehealth use may introduce omitted variable bias, estimated associations may be attenuated or inflated, depending on whether these factors act as confounders or mediators, and should therefore be interpreted with caution. Third, some measures, such as visit type and service type, rely on coding in MEPS, which may not capture all nuances of telehealth encounters. Because service-type categories in MEPS are broadly defined, hybrid or multi-purpose encounters may be misclassified, which could affect interpretation of service-specific findings. Fourth, the MEPS definition of telehealth does not capture some kinds of telehealth visits that may be available to patients, including hybrid, remote patient monitoring, and asynchronous modalities such as text-based messaging. This definition is quite common in the literature [7,11,19,20,21]. However, it is likely that the measure primarily captures synchronous care and omits asynchronous care. As a result, our estimates underestimate the utilization of all health care that is not face-to-face. Fifth, annual aggregation of telehealth use may mask substantial intra-year volatility, particularly during the early pandemic period when telehealth adoption changed rapidly. For example, sharp increases observed in spring 2020 are averaged over the full year in our analyses, resulting in lower annual rates. Accordingly, our regression estimates should be interpreted as reflecting year-to-year average differences, rather than short-term responses to policy or public health shocks. In addition, differential survey nonresponse during the COVID-19 pandemic may introduce selection bias if marginalized populations were less likely to participate; although MEPS weights partially adjust for nonresponse, residual bias may remain. Lastly, because MEPS excludes institutionalized populations, the findings may not be generalizable to these groups and may underestimate telehealth utilization among individuals with very high levels of use, such as patients receiving intensive mental health services. Despite these limitations, the use of nationally representative data allows for a broad understanding of telehealth trends and associated factors in the U.S., providing valuable insights for policymakers and health care systems.

## 5. Conclusions

Using nationally representative data from MEPS from 2020 to 2023, this study found that telehealth utilization increased sharply during the early pandemic and has remained relatively stable since. Telehealth visits were primarily outpatient and most commonly involved mental health care. Demographic and socioeconomic factors—including sex, age, health insurance status, and family income—were significant predictors of telehealth use, highlighting persistent disparities in access. These findings indicate that some pandemic-related gains in telehealth have endured, potentially improving access to care for certain populations. Continued support from health care systems and policymakers is essential to sustain telehealth services, promote equitable access, and address disparities in health care delivery.

## Figures and Tables

**Table 1:** Population-level visit utilization, 2020–2023.

	2020	2021	2022	2023	*p*-Value
N	328,545,297 (24.8%)	331,249,393 (25.0%)	333,053,243 (25.1%)	334,530,273 (25.2%)	
Average number of visits per person (office + outpatient)	6.8 (13.6)	7.7 (14.6)	7.1 (13.8)	7.6 (14.1)	<0.001
Average number of telehealth visits per person (office + outpatient)	0.3 (2.0)	0.7 (4.3)	0.7 (4.3)	0.7 (4.1)	<0.001

Note: All numbers are weighted to reflect survey weights.

**Table 2:** Annual visit-level telehealth rates and person-level telehealth use, 2020–2023.

Year	Total Visits (Millions)	Telehealth Visits (Millions)	Telehealth Rate (% of Visits)	95% CI		Proportion of Population with ≥1 Telehealth Visit (%)
2020	2230.1	90.4	1.8%	1.7%	2.0%	7.2%
2021	2563.0	246.8	4.5%	4.1%	4.8%	13.2%
2022	2372.8	230.0	4.2%	3.8%	4.5%	12.0%
2023	2531.0	245.2	4.4%	3.9%	4.9%	12.1%

Note: Telehealth rate represents the proportion of office-based and outpatient visits delivered via telehealth. The proportion of the population with ≥1 telehealth visit represents the percentage of individuals with at least one telehealth visit during the year. All estimates are weighted to reflect the U.S. civilian, noninstitutionalized population. Visit counts are rounded to the nearest 0.1 million to reduce spurious precision.

**Table 3:** Visit-level telehealth rates and person-level telehealth use by visit type (office-based vs. outpatient), 2020–2023.

Year	Office Visits (Millions)	Telehealth Office Visits (Millions)	Telehealth Rate (% of Office Visits)	Proportion of Population with ≥1 Office Telehealth Visit (%)	Outpatient Visits (Millions)	Telehealth Outpatient Visits (Millions)	Telehealth Rate (% of Outpatient Visits)	Proportion of Population with ≥1 Outpatient Telehealth Visit (%)
2020	1986.2	71.1	1.9%	4.9%	243.9	19.2	8.6%	2.7%
2021	2271.4	200.3	4.5%	9.3%	291.6	46.5	15.2%	5.2%
2022	2077.9	190.2	4.5%	8.3%	294.9	39.8	15.2%	4.9%
2023	2214.2	203.7	4.8%	8.6%	316.8	41.5	12.7%	4.5%

Note: Telehealth rates represent the proportion of visits delivered via telehealth within each visit type. Proportions of the population represent the percentage of individuals with at least one telehealth visit in the specified setting during the year. All estimates are weighted to reflect the U.S. civilian, noninstitutionalized population. Visit counts are rounded to the nearest 0.1 million to reduce spurious precision.

**Table 4:** Visit-level telehealth rates by service type, 2020–2023.

Visit Type	2020 Telehealth Rate	2021 Telehealth Rate	2022 Telehealth Rate	2023 Telehealth Rate
Check-up	0.9%	1.9%	1.4%	1.3%
Diagnosis	0.9%	2.1%	2.2%	1.9%
Emergency	0.0%	0.1%	0.1%	0.1%
Psychotherapy	1.2%	3.8%	3.5%	4.2%
Follow-up	0.9%	1.9%	1.8%	1.5%
Other	0.2%	0.5%	0.4%	0.5%

Note: Telehealth rates represent the proportion of visits delivered via telehealth within each service type. Service types are defined using MEPS visit-level categories and may include hybrid or multi-purpose encounters. All estimates are weighted to reflect the U.S. civilian, noninstitutionalized population and are rounded to reduce spurious precision.

**Table 5:** Two-part model results for telehealth utilization.

	Binary Hurdle Model (Logistic Regression)		Negative Binomial Model	
Variable	Odds Ratio (OR)	*p*-Value	Incidence Rate Ratios (IRR)	*p*-Value
Year				
2020	Reference		Reference	
2021	2.02	<0.01	1.39	<0.01
2022	1.86	<0.01	1.36	<0.01
2023	1.81	<0.01	1.50	<0.01
Age category				
0–17	Reference		Reference	
18–64	2.33	<0.01	1.0	0.50
≥65	1.81	<0.01	0.5	<0.01
Female	1.37	<0.01	1.0	0.31
Race/Ethnicity				
Non-Hispanic White	Reference		Reference	
Hispanic	0.83	<0.01	1.0	0.27
Non-Hispanic Black	0.77	<0.01	1.1	0.03
Non-Hispanic Asian	0.81	0.01	0.9	0.11
Non-Hispanic others	1.02	0.88	1.0	0.96
Family income as % of poverty line				
Poor	Reference		Reference	
Near poor	1.02	0.84	0.9	0.45
Low income	1.06	0.37	1.0	0.48
Middle income	1.11	0.08	0.9	0.15
High income	1.35	<0.01	0.9	0.12
Insurance type				
Private	Reference		Reference	
Public (Medicaid and Medicare)	1.00	0.95	1.0	0.11
Uninsured	0.31	<0.01	1.0	0.78
Number of office and outpatient visits	1.05	<0.01	1.04	<0.01

Note: The binary hurdle model estimates the probability of any telehealth use and reports odds ratios (ORs). The negative binomial model estimates the number of telehealth visits among individuals with at least one telehealth visit and reports incidence rate ratios (IRRs). All models are survey-weighted and account for the MEPS complex sampling design. Estimates are rounded to reduce spurious precision.

## Data Availability

The original data presented in the study are openly available in FigShare Version 2 at 10.6084/m9.figshare.30777665 (accessed on 3 December 2025).

## Appendix A

**Table A1:** Weighted Demographic Characteristics of the MEPS Population, 2020–2023.

	2020	2021	2022	2023	*p*-Value
Age category					0.602
0–17	22.1%	22.1%	21.6%	21.6%	
18–64	60.2%	60.2%	60.3%	60.0%	
≥65	17.7%	17.7%	18.1%	18.4%	
Sex					0.772
Male	49.0%	49.2%	49.3%	49.3%	
Female	51.0%	50.8%	50.7%	50.7%	
Race/Ethnicity					0.714
Hispanic	18.8%	19.0%	19.3%	19.7%	
Non-Hispanic White	59.2%	58.6%	57.9%	57.4%	
Non-Hispanic Black	12.5%	12.4%	12.5%	12.5%	
Non-Hispanic Asian	6.0%	6.1%	6.3%	6.4%	
Non-Hispanic others	3.6%	4.0%	4.0%	4.1%	
Family income as % of poverty line					0.893
Poor	11.5%	11.5%	11.5%	11.1%	
Near poor	3.8%	4.0%	3.8%	3.8%	
Low income	12.4%	12.1%	12.4%	12.1%	
Middle income	28.3%	28.4%	29.6%	28.7%	
High income	44.1%	43.9%	42.7%	44.3%	
Insurance type					0.227
Private	66.2%	66.1%	65.2%	64.5%	
Public (Medicaid and Medicare)	27.3%	27.8%	28.3%	29.1%	
Uninsured	6.5%	6.1%	6.4%	6.3%	

## Appendix B

**Table A2:** Visit-Level Telehealth Rates by Demographic Characteristics, 2020–2023.

Variable	2020	2021	2022	2023
Age group				
0–17	1.4%	3.2%	2.3%	2.1%
18–64	2.0%	5.2%	5.2%	5.8%
≥65	1.9%	3.6%	3.0%	2.5%
Sex				
Male	1.6%	3.9%	3.5%	4.1%
Female	2.1%	5.0%	4.8%	4.7%
Race/ethnicity				
Hispanic	1.8%	4.1%	3.2%	3.2%
Non-Hispanic White	2.0%	4.7%	4.7%	5.1%
Non-Hispanic Black	1.6%	3.7%	3.6%	4.0%
Non-Hispanic Asian	1.7%	4.2%	3.7%	3.3%
Non-Hispanic others	1.7%	5.2%	3.5%	4.5%
Family income as % of poverty line				
Poor	1.9%	4.3%	3.6%	3.5%
Near poor	1.6%	4.5%	3.3%	2.8%
Low income	1.9%	3.8%	3.1%	3.2%
Middle income	1.7%	4.3%	3.6%	4.2%
High income	2.0%	4.8%	5.1%	5.3%
Insurance type				
Private	1.8%	4.6%	4.9%	5.3%
Public (Medicaid and Medicare)	2.2%	4.6%	3.2%	3.0%
Uninsured	0.6%	1.8%	1.5%	2.0%

Note: Telehealth rates represent the proportion of visits delivered via telehealth within each demographic group. Estimates are based on office-based and outpatient visits, weighted to reflect the U.S. civilian, noninstitutionalized population, and rounded to reduce spurious precision.
